# Initial experience of robot-assisted thoracoscopic surgery in China

**DOI:** 10.1002/rcs.1589

**Published:** 2014-04-29

**Authors:** Jia Huang, Qingquan Luo, Qiang Tan, Hao Lin, Liqiang Qian, Xu Lin

**Affiliations:** 1 Clinical Centre for Pulmonary Tumour, Shanghai Jiaotong University Affiliated Shanghai Chest HospitalPeople's Republic of China; 2 Department of Thoracic Surgery, Jiangsu Cancer HospitalPeople's Republic of China

**Keywords:** robot-assisted thoracoscopic surgery (RATS), video-assisted thoracoscopic surgery (VATS), da Vinci surgical system, thoracic surgery, pulmonary adenocarcinoma

## Abstract

**Background:**

The objective of this study was to evaluate the safety and feasibility of robot-assisted thoracoscopic surgery (RATS).

**Methods:**

From May 2009 to May 2013, 48 patients with intrathoracic lesions underwent RATS with the da Vinci® Surgical System was reported (11 lobectomies, 37 mediastinal tumour resections).

**Results:**

RATS was successfully and safely completed in all 48 patients. Conversion of the operation to open surgery was not needed in any patient. The average operation time was 85.9 min, average blood loss 33 ml, and average hospital stay 3.9 days. No patient required blood transfusion. The only recognized adverse event was the development of a bronchopleural fistula in one patient.

**Conclusions:**

RATS appears feasible and safe in thoracic surgery. More investigation will be needed in order to determine its possible long-term benefits and cost effectiveness. Copyright © 2014 John Wiley & Sons, Ltd.

## Introduction

Video-assisted thoracoscopic surgery (VATS) is a minimally invasive procedure, which is being adopted for use in thoracic oncology 1,2. VATS for lobectomy is much less traumatic than thoracotomy, as it avoids division of the chest wall muscles and rib spreading, thus resulting in less pain, shorter duration of chest-tube drainage, and shorter hospital stay 3,4. Standard VATS instruments are rigid and limit the operator's freedom of movement 5. Furthermore, visualization of the operating field is flat and only two-dimensional. Thus, robot-assisted thoracoscopic surgery (RATS) is being developed in order to overcome the limitations of VATS. The high-definition, three-dimensional (3D) image of the da Vinci® Surgical System (Intuitive Surgical, Mountain View, CA, USA) facilitates the identification and dissection of anatomical structures during operation. The wide range of motion of the multi-articulated instruments permits complex suturing and intracorporal knot tying; tremor filtration and motion scaling allow for more precise movements than are possible with VATS 6–9.

The da Vinci system consists of a console and surgical arm cart, a manipulator unit with several arms (called the patient cart) and a 3D, high-definition vision cart. The operative instruments are introduced into the chest via special ports and are attached to the arms of the robot. The system can downscale from the motions of the handles to that of the surgical instruments from 3:1. The greatest benefit of the robotic technique is its ability to operate in remote areas of the chest that are difficult to reach. The robotic procedures usually are performed by two surgeons (one positioned on the console and one at the tableside). The objective of this study was to evaluate the feasibility and safety of RATS.

## Patients and methods

### Patients

We retrospectively reviewed our initial 48 patients with intrathoracic lesions who underwent RATS with the da Vinci robotic system during May 2009–May 2013. Informed consent was obtained from all patients, and the study was approved by our hospital's Institutional Ethics Committee on Human Research. Table1 summarizes the intrathoracic locations of the lesions, the surgical access route and the number of trocars used. The study included 24 male and 24 female patients, mean age 50.6 (range 28–66) years. Eleven of the 48 patients had suspected lung cancer; one of these patients had the middle lobe syndrome. Of 37 mediastinal lesions, 32 were located in the anterior mediastinum and five in the posterior mediastinum.

**Table 1:** Location of lesions

Location	Number
Lower right lobe	3
Middle right lobe	2
Upper anterior mediastinum	32
Upper left lobe	3
Upper posterior mediastinum	5
Upper right lobe	3

Inclusion criteria for lobectomy were: peripheral nodule, tumour diameter ≤ 3 cm, well-developed lobar fissure, no extensive pleural adhesions, diameter of interlobar lymph node < 1 cm without apparent calcified lymph nodes, and heart and lung function suitable for general anaesthesia and single-lung ventilation by preoperative evaluation. Patients with central lung cancer were excluded.

Inclusion criteria for mediastinal tumours were: tumour size < 5 cm and clear boundaries between tumours, large blood vessels without signs of extrapulmonary invasion, and heart and lung function suitable for general anaesthesia and single-lung ventilation by preoperative evaluation.

Clinical endpoints of this study were blood loss, postoperative hospital stay, start-up time, console time, total operation time and postoperative complications.

### Surgical techniques

#### Robot-assisted lobectomy

**Figure 1:**
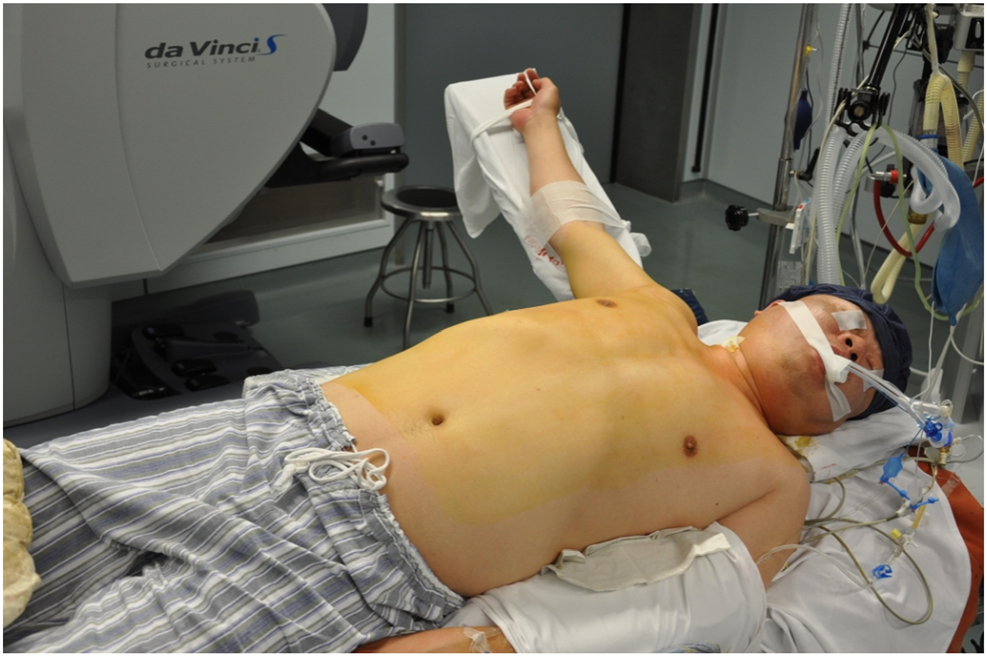
The patient was placed in the decubitus position with the right side tilted up 45° and the right arm abducted

The patients were placed in the lateral decubitus position for a posterolateral incision. To facilitate the placement of the sleeve tube, the operating table was tilted slightly, thus widening the chest's intercostal space. Under general anaesthesia, patients were placed on double-lumen tracheal intubation and contralateral single-lung ventilation. The da Vinci system was placed near the cephalic side of the patient, forming a 15° angle with the patient's longitudinal axis. The robotic arm remained vertical to the cart's latitudinal line. A cannula was placed in a different location from the thoracoscopy location (Figure1). A service port was created in the seventh intercostal space in the mid-axillary line through a 1 cm incision. A sleeve tube, then the robotic thoracic endoscope, was inserted through the incision. With guidance by thoracic endoscopy, two more 1 cm incisions were created (at the seventh intercostal space in the mid-axillary line and in the posterior axillary line) for introduction of the robotic arms into the chest cavity. An auxiliary port was placed at the fourth intercostal space in the anterior axilla, as an entrance site for Endo-staplers, suctioning and retrieval of specimens. The catheters were spaced about 9–10 cm apart in order to prevent mechanical collision and instrument damage. The lung was deflated through CO_2_ release. A harmonic scalpel was held by the right robotic arm and a Cadiere clamp (Intuitive Surgical) by the left arm, thus permitting sufficient vision and exposure of the surgical field. There were 11 cases who underwent lobectomy, among which eight cases were confirmed as adenocarcinoma and received lymph node dissection.

#### Anterior mediastinal thymectomy

**Figure 2:**
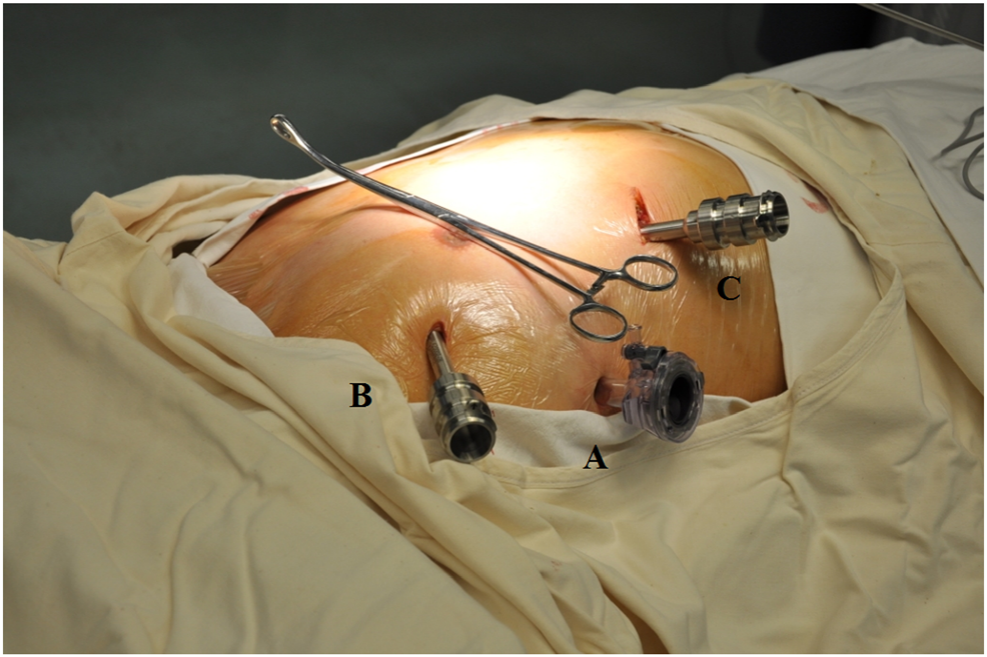
Positioning of ports: (A) the robotic endoscope port at the right sixth intercostal space in the anterial axillary line; (B) right instrument port at the right third intercostal space in the anterior axillary line; (C) left instrument port at the right sixth intercostal space in the midclavicular line. The forceps are shown pointing to the thymoma and the axis of the da Vinci cart

**Figure 3:**
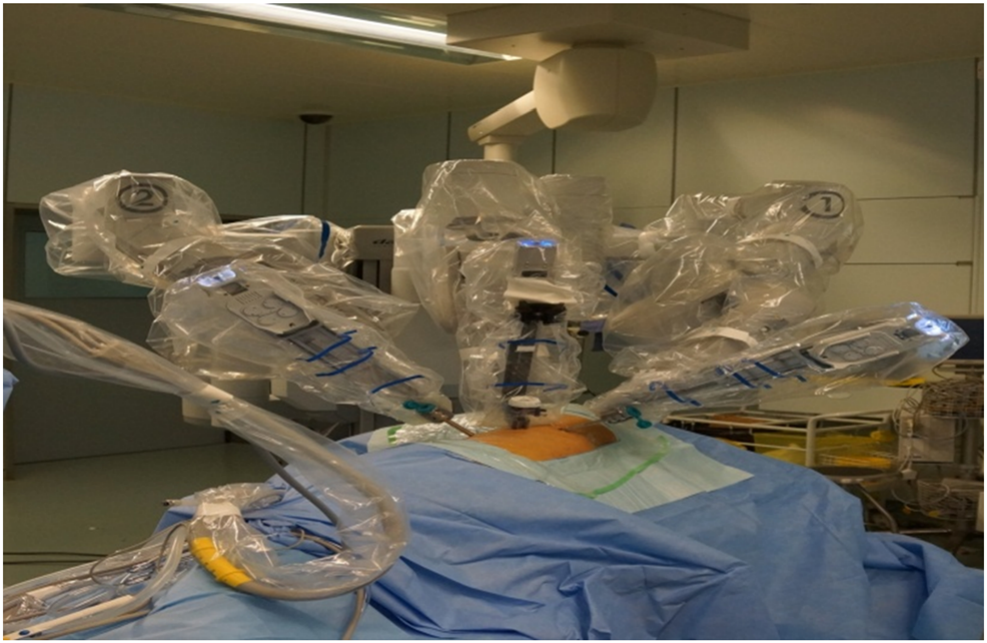
Positioning of ports (more distant view)

Patients were placed supine, with their right side elevated 45° and their right arm abducted at 90°. The robot cart was located on the patient's left side. The central axis, the light-source port and the thymoma were kept in a projection line on the patient's thoracic surface. The light-source port was located at the right sixth intercostal space in the anterior axillary line. A thoracoscope was introduced into the chest cavity in order to determine the location and extent of the tumour, the extent of pleural adhesions and the degree of ipsilateral lung deflation. Under thoracoscopic guidance, two manipulating ports were created, at the right third intercostal space in the anterior axillary line and at the right sixth intercostal space in the midclavicular line. The operating port was placed 10 cm away from the light source port (Figure2). An electrocoagulation hook was held by the robot's right arm and a Cadiere clamp by the left arm, thus enhancing the visual field. Along the ventral side of the superior vena cava, using the electric coagulation hook, we made a lengthy incision on the mediastinal pleura, from the junction of the left and right brachiocephalic veins down to the reflection of the pericardium. When the mediastinal fat had been dissected, we first located and exposed the contralateral phrenic nerve. Most of the time the contralateral pleurae were broken, but this did not increase the incidence of postoperative complications. We then mobilized the inferior pole of the anterior mediastinum along the phrenic nerve, and bluntly dissected the tumour from the mediastinal adipose tissues. Next, we gently lifted the tumour and bluntly dissected it from the contralateral pleural reflection (internal thoracic artery) retrosternally. We further mobilized the thymoma from its lower contralateral superior pole with sharp dissection and control of bleeding with electric coagulation. Figure3

Extensive exposure of the brachiocephalic veins, located in the dorsal superior pole of the tumour, was usually necessary in order to avoid injury that might necessitate open surgery. Arterial branches supplying the tumour were ligated with Hem-O-lock® or were endoscopically ligated. Finally, the entire thymoma and associated mediastinal adipose tissues were removed from the chest through the right operating port. Dissection of the adipose tissue was performed, followed by sequential removal of the thymoma from the superior vena cava, right phrenic nerve, pericardium and contralateral phrenic nerve.

#### Posterior mediastinal tumour resection

For this operation, the light-source port was located in the anterior axillary line at various intercostal spaces, depending on the location of the tumour. The other two operating ports were located 9–10 cm away from the light source, between the left and right light-source holes. The right hand of the operator performed sharp dissection, while the left hand held a Cadiere clamp in order to obtain good exposure of the surgical field.

## Results

### Perioperative outcome

Forty-eight patients were successfully and safely operated upon with RATS. The type of surgery performed, blood loss, duration of hospital stay, start-up time, console time, total operation time (from the skin incision to wound closure) and histopathological diagnoses of the resected lesions are given in Table2. None of the RATS procedures required conversion to an open operation. The average operation time was 85.9 min, average blood loss 32.9 ml and average hospital stay 3.9 (range 2–31) days. Only one surgical complication, a bronchopleural fistula, was encountered; the fistula was repaired on postoperative day 16, and the patient was discharged fully recovered.

**Table 2:** Peri-operative outcome

Type of surgery	Number (total = 48)	Blood loss (ml)	Hospital stay (days)	Start-up time (min)	Console time (min)	Total operation time (min)	Follow-up (months)
Excision of bronchial cyst	3	10.0	2.3	15.0	55.0	81.7	8.3
Excision of epidermal cysts	1	10.0	2.0	15.0	60.0	80.0	7.0
Excision of mediastinal ganglion cell tumour (posterior mediastinum)	1	10.0	2.0	18.0	40.0	65.0	8.0
Excision of mediastinal neurogenic tumour	1	60.0	4.0	66.0	75.0	95.0	47.0
Excision of mediastinal neurogenic tumour (posterior mediastinum)	1	10.0	2.0	20.0	45.0	75.0	9.0
Excision of synovial sarcoma (posterior mediastinum)	1	10.0	2.0	17.0	40.0	63.0	10.0
Excision of thymic cyst	1	60.0	2.0	50.0	79.0	104.0	48.0
Lower right lobectomy + systematic lymphadenectomy	4	19.1	1.5	17.9	46.5	46.0	18.7
Middle right lobectomy + partial upper right lobe posterior segment wedge resection	1	40.0	5.0	70.0	90.0	100.0	45.0
Middle right lobectomy + partial upper right lobe wedge resection	1	60.0	8.0	60.0	150.0	168.0	12.0
Middle right lobectomy + partial upper right posterior segment wedge resection	1	50.0	4.0	55.0	100.0	113.0	45.0
Thymectomy	16	13.1	0.8	10.1	31.3	31.1	9.4
Thymectomy, extended	12	18.8	2.4	19.7	42.0	62.4	10.3
Upper right or left lobectomy + systematic lymphadenectomy	4	62.5	12.3	39.5	121.3	136.3	33.3

### Follow-up and survival

Patients were followed up for an average of 16 (range 1–48) months. One patient died of stage IIIa lung cancer 14 months after surgery; all others survived.

## Discussion

The major result of this study, the first recorded experience with RATS in China, was that 48 patients operated upon with this newly developed procedure had a technically successful outcome, with only one significant adverse event. Eight of the patients had a lobectomy for lung cancer and all have survived, except for one who died after 14 months from recurrent of stage IIIa cancer. The use of minimally invasive surgery in the treatment of lung cancer has been controversial because of uncertainty about whether the operation is safe and can achieve complete resection of the cancers. Our experience indicates that the minimally invasive RATS approach to lung cancer, and other thoracic conditions as well, has considerable promise.

Thus far, VATS has been the favoured procedure for minimally invasive thoracic operations. However, VATS has certain disadvantages, some of which may be overcome with RATS. Compared with VATS, RATS provides 3D, high-definition dynamic visualization. Its unique eye-ball tracker can accurately track a surgeon's moving vision, converting dynamic imaging to static imaging, a feature that permits the operator to see the static imagine when the robotic instrument is moving in a patient's body. Furthermore, the EndoWrist® in the da Vinci system is superior to the human wrist, as it is flexible in all directions. The robotic system is also superior to open surgery in at least one respect: it enables a wider range of joint motion in the endoscopic component of the operation and full coordination between the operator's hands and eyes; instead of standing to the side operating table, the surgeon can sit in a console away from the patient through the entire operation. Ergonomics for the surgeon, working on a lengthy operation, are much better than in traditional laparoscopic surgery. Surgeons who work with a robot usually have a short learning curve 10–12. The system limits the range and direction of hand movement; moreover, the operator's hand movement and the equipment movement are in opposite directions, and the trocars act as the fulcrum of a lever effect, which may produce substantial shear force, causing tissue damage and increase in the operator's fatigue. Thus, despite its advantages, traditional VATS poses some technical and safety challenges, especially in radical resection and systematic lymph node dissection in lung cancer.

In response to these challenges, a new generation of minimally invasive surgical techniques is emerging. Robot-assisted operating systems (RATS) are one such advance. RATS and VATS can be used complementarily in the dissection of fine structures, such as veins and bronchial and mediastinal lymph nodes. Many studies have found no difference between VATS and open surgery in the number of lymph nodes dissected, but Sagawa *et al.* 13 reported that VATS missed at least 3% of lymph nodes. Our experience with RATS in the thorax has shown that the upper mediastinal fatty and lymphoid tissue can be entirely resected; the clearer vision and finer movements with EndoWrist permit more thorough dissection of lymph nodes than can be achieved with open surgery.

With its delicate robotic arms, RATS can ligate blood vessels thoracoscopically as easily as VATS does, with greatly reduced cost of endoscopic surgical instruments. Our experience has shown that RATS is an efficacious and safe minimally invasive approach to the resection of early-stage lung carcinoma. RATS is particularly helpful in the dissection of lymph nodes. The efficacy and benefit of RATS in lung cancer surgery should be established through multicentre randomized clinical trials.

The da Vinci system also appears well suited for the resection of mediastinal tumours. We completed 32 operations for such tumours, with no postoperative complications or recurrences. Some patients complained of mild postoperative chest pain and shoulder discomfort, but these symptoms resolved spontaneously after 3 months. We suspect that the pain and discomfort were due to mild trocar-induced trauma to the intercostal nerves.

The nimble robot wrists allow surgeons to readily complete a variety of actions inside the narrow chest cavity. Especially in the case of thymomas, mobilization of the superior pole of the tumour is technically difficult and dangerous, as injury to the brachiocephalic veins would lead to massive bleeding. However, the use of RATS decreased the risk significantly. Thymoma resection with VATS can only be done in a single direction, so extending the mediastinal and neck fat tissue dissection is technically difficult; however, with RATS, both the contralateral and para-diaphragmatic fat tissue can be completely dissected 14,15. Because of the malignant potential of thymoma, we have performed extensive tumour resections in eight patients. In the operation, we accessed the thymoma via the right chest so that the dissection could be extended up to the edge of the left phrenic nerve. Complete removal of the adjacent adipose tissue may reduce the risk of tumour recurrence. VATS thymectomy has become an increasingly attractive option because of its better intra-thorax visualization and reduced trauma. For patients who have myasthenia gravis due to thymoma, traditional thymectomy may cause greater surgical trauma; the use of minimally invasive surgery has reduced the frequency and severity of this complication. However, due to the limited space in the anterior mediastinum and inflexibility of the VATS equipment, operative manoeuvrability with this approach is restricted. Furthermore, the artificial pneumothorax and sternum elevation may worsen the surgical trauma to brachiocephalic veins and other large vessels 16. These problems are avoided with RATS. Radical removal of adipose tissues may reduce the risk of myasthenia gravis recurrence. Thus, we feel that RATS technology is worthy of large-scale application in the treatment of thymomas in myasthenia gravis.

With regard to the da Vinci equipment for the removal of anterior mediastinal tumours, three ports and tumour projection on the thoracic surface should form a diamond-shaped area, with two robotic arm ports located on one of the diagonals. The endoscopic port and the tumour projection form another diagonal. The robot axis is also located on the extension of the diagonal line. The space between the two robotic arm ports and the endoscopic port should be about 10 cm (about the width of a palm), which can prevent their colliding during the operation. The endoscopic port is best set at the anterior axillary line. With this arrangement, both the anterior chest wall and the sternum can be lifted up with the robot arm, so more space is available in the anterior mediastinum to facilitate visualization and exposure.

As for resection of posterior mediastinal tumours, we think that the use of the da Vinci system facilitates their complete removal 17,18. Removal of a well-capsulated tumour with a clear boundary is relatively simple and straightforward, so RATS may not be superior to VATS in this instance. However, for operation on a tumour located close to the apex of the chest wall, with an ill-defined edge interfaced with surrounding nerves and vessels, RATS may be the better option. In one of our patients, the tumour was located near the right lung apex, with an ill-defined edge interfaced with the brachial plexus. Based on our previous experience with VATS, with its limited 2D vision, we expected the risk of bleeding to be a major challenge. However, RATS provides high-resolution 3D imaging and × 10 vision magnification, so we could easily distinguish the tumour from blood vessels and nerves.

Although the da Vinci surgical system has many advantages, it also has some deficiencies. Due to the lack of tactile feedback, the operator cannot well control the force exerted in mobilizing blood vessels away from other organs, so damage to blood vessels is hard to avoid. Because of this tactile deficit, the traditional thoracicoscopic cutter must be used on the trachea, even though the EndoWrist is nimbler than human hands. When the cutter is used, the mechanical robotic arm usually has to be moved out of the body to make way for the cutter. The operation then incurs greater risk, operation time and hospitalization cost. In addition, a fourth auxiliary port must be made for the insertion of suction devices and retractors, which are needed for exposing the surgical field. In the entire operation, one of the assistants has to ceaselessly exchange surgical instruments according to the surgeons' request, e.g. withdrawing the robotic arm, inserting the endoscopic cutter (Echelong 60, Johnson &Johnson USA) and then re-entering the body.

The high cost of RATS is the main reason why it is not widely accepted and applied 19. Our da Vinci RATS system cost more than $US 3 000 000. The ongoing costs include those of system maintenance and upgrades and the purchase of special instruments and consumables. In China, RATS costs about $US 3000 more than the traditional VATS; we believe that the superior technology of RATS is worth the modestly higher cost.

In our initial experience, the robotic system has some technical advantages over VATS, e.g. excellent 3D, high-definition vision and fine operation of its hands, which facilitate dissection of lymph nodes and mediastinal adipose tissues. However, performing a multicentre randomized trial to determine efficacy of RATS vs VATS on lymphadenectomy is not practically feasible. More feasible approaches may be large-sample observational studies (either cohort or case-control study) in the future.

In conclusion, RATS appears feasible and safe in thoracic surgery. More investigation will be needed in order to determine its possible long-term benefits and cost effectiveness.

## Conflict of interest

The authors have stated explicity that there are no conflicts of interest in connection with this article.

## Funding

No specific funding.

## References

[b1] Thomas P, Doddoli C, Yena S (2002). VATS is an adequate oncological operation for stage I non-small cell lung cancer. Eur J Cardiothorac Surg.

[b2] Roviaro GC, Varoli F, Vergani C (2002). State of the art in thoracospic surgery: a personal experience of 2000 videothoracoscopic procedures and an overview of the literature. Surg Endosc.

[b3] Nagahiro I, Andou A, Aoe M (2001). Pulmonary function, postoperative pain, and serum cytokine level after lobectomy: a comparison of VATS and conventional procedure. Ann Thorac Surg.

[b4] Förster R, Storck M, Schäfer JR (2002). Thoracoscopy versus thoracotomy: a prospective comparison of trauma and quality of life. Langenbecks Arch Surg.

[b5] Dieter RA, Kuzycz GB (1997). Complications and contraindications of thoracoscopy. Int Surg.

[b6] Onnasch JF, Schneider F, Falk V (2002). Five years of less invasive mitral valve surgery: from experimental to routine approach. Heart Surg Forum.

[b7] Nifong LW, Chu VF, Bailey BM (2003). Robotic mitral valve repair: experience with the da Vinci system. Ann Thorac Surg.

[b8] Tewari A, Peabody J, Sarle R (2002). Technique of da Vinci robot-assisted anatomic radical prostatectomy. Urology.

[b9] Giulianotti PC, Coratti A, Angelini M (2003). Robotics in general surgery: personal experience in a large community hospital. Arch Surg.

[b10] Schurr MO, Arezzo A, Buess GF (1999). Robotics and systems technology for advanced endoscopic procedures: experiences in general surgery. Eur J Cardiothorac Surg.

[b11] Eadie LH, Seifalian AM, Davidson BR (2003). Telemedicine in surgery. Br J Surg.

[b12] Schmid T (2002). Editorial to: main topics: robotic surgery. Eur Surg.

[b13] Sagawa M, Sato M, Sakurada A (2001). A prospective trail of systematic nodal dissection for lung cancer by video-assisted thoracic surgery: can it be perfect?. Ann Thorac Surg.

[b14] Yim AP (1997). Thoracoscopic thymectomy: which side to approach?. Ann Thorac Surg.

[b15] Mack MJ (2001). Video-assisted thoracoscopy thymectomy for myasthenia gravis. Chest Surg Clin N Am.

[b16] Maat AP, Van Doorn PA, Bogers AJ (2008). Inclusion of the transcervical approach in the video-assisted thoracoscopic extended thymectomy (BATET) for myasthenia gravis: a prospective trial. Surg Endosc.

[b17] Kumar A, Kumar S, Aggarwal S (2002). Thoracoscopy: the preferred approach for the resection of selected posterior mediastinal tumors. J Laparoendosc Adv Surg Tech.

[b18] Bodner J, Wykypiel H, Wetscher G (2004). First experiences with the da Vinci operating robot in thoracic surgery. Eur J Cardiothorac Surg.

[b19] Lanfranco AR, Castellanos AE, Desai JP (2004). Robotic surgery: a current perspective. Ann Surg.

